# Trail Making Test performance contributes to subjective judgment of visual efficiency in older adults

**DOI:** 10.7717/peerj.1407

**Published:** 2015-12-07

**Authors:** Annalisa Setti, James Loughman, George M. Savva, RoseAnne Kenny

**Affiliations:** 1 The Irish Longitudinal Study on Ageing (TILDA), Trinity College Dublin, Dublin, Ireland; 2 School of Applied Psychology, University College Cork, Cork, Ireland; 3 Optometry Department, College of Sciences and Health, Dublin Institute of Technology, Dublin, Ireland; 4 African Vision Research Institute, Faculty of Sciences and Health, University of KwaZulu Natal, Durban, South Africa; 5 Mercer’s Institute for Successful Ageing, St. James Hospital, Dublin, Ireland

**Keywords:** Self-report, Visual acuity, Ocular pathology, Trail making test, Visual search

## Abstract

**Introduction.** The determinant factors that influence self-reported quality of vision have yet to be fully elucidated. This study evaluated a range of contextual information, established psychophysical tests, and in particular, a series of cognitive tests as potentially novel determinant factors.

**Materials & Methods.** Community dwelling adults (aged 50+) recruited to Wave 1 of The Irish Longitudinal Study on Ageing, excluding those registered blind, participated in this study (*N* = 5,021). Self-reports of vision were analysed in relation to visual acuity and contrast sensitivity, ocular pathology, visual (Choice Response Time task; Trail Making Test) and global cognition. Contextual factors such as having visited an optometrist and wearing glasses were also considered. Ordinal logistic regression was used to determine univariate and multivariate associations.

**Results and Discussion.** Poor Trail Making Test performance (Odds ratio, OR = 1.36), visual acuity (OR = 1.72) and ocular pathology (OR = 2.25) were determinant factors for poor versus excellent vision in self-reports. Education, wealth, age, depressive symptoms and general cognitive fitness also contributed to determining self-reported vision.

**Conclusions.** Trail Making Test contribution to self-reports may capture higher level visual processing and should be considered when using self-reports to assess vision and its role in cognitive and functional health.

## Introduction

Many subjective psychological constructs have been shown to have a number of objective underlying determining factors. Self reported memory is a clear example, in so much that it has been argued that other psychological aspects such as depression and personality could be more linked to self reports of memory than memory itself (Abdulrab & Heun, 2008). In the present work, we analyzed another psychological construct, self reported vision, assessed by a simple question “How would you rate your vision? Excellent, Very good, Good, Fair or Poor?” and investigated the contribution of cognitive factors to this construct in a large sample of individuals aged 50 and over.

Although self reported vision is clearly associated with visual acuity, it is also clear that the answer to one simple question on one’s own visual ability can be largely dependent on factors other than visual acuity. The specific factor investigated here was visual processing, assessed by the Choice Response Time task and the Trail Making Test tasks. Whether impairment in visual processing can be captured by subjective reports of poor vision independently from global cognitive impairments and lower level acuity impairment (more related to optical factors) remains to be determined and could contribute in explaining why some individuals with good visual acuity report poorer vision and vice versa. In the domain of hearing, extensive work has been devoted to central presbycusis showing that specific hearing deficits linked to central hearing processing may exist independently both from peripheral deficits and central cognitive deficits, and can be captured in the subjective experience of individuals with poor hearing (Humes et al., 2012).

With ageing, the relationship between sensory efficiency and cognitive performance becomes closer (Lindenberger & Baltes, 1994; Lindenberger & Ghisletta, 2009) and self reports of vision are predictive in terms of negative cognitive outcomes such as dementia (Rogers & Langa, 2010). While it is established that visual acuity is not the only determinant of self reported vision (Rubin et al., 2001), it is necessary to understand what factors underlie it. Among these factors, arguably, the least investigated is the cognitive dimension, while greater emphasis has been given to objective visual measures such as visual acuity (Klein et al., 1999; Rubin et al., 2001), use of vision to accomplish daily tasks (e.g., Fors, Thorslund & Parker, 2006; Kempen et al., 1996; Rubin et al., 2001; Rubin et al., 2007), and socio-demographic factors (El-Gasim et al., 2012; Klein et al., 1999; Rubin et al., 2001).

Among cognitive factors, previous studies have investigated intelligence (Kempen et al., 1996) and global cognitive status (Hidalgo et al., 2009), but their results are limited by the impossibility of jointly controlling for a range of confounding factors known to influence self reports, such as socio-economic status, depression, or global health status (e.g., Dufouil, Fuhrer & Alpérovitch, 2005; Ponds, Van Boxtel & Jolles, 2000) which we can control for in the present study. Importantly to our aim, whether specific cognitive tasks requiring visual processing are associated with self reported vision at a population level has not been studied, despite the fact that self reports are used in different epidemiological studies (e.g., the English Longitudinal Study on Ageing, UK and the Health and Retirement Study, USA). Further our understanding of the underlying cognitive factors to self reported vision could also inform the use of self reports in clinical settings, where knowing the validity of a simple question could inform on the need for further assessments that go beyond a visual acuity test (Table S1 provides an overview of studies including socio-demographic and cognitive determinants of self reported vision).

Measures of visual processing differ from traditional psychophysical measures such as visual acuity, particularly in relation to the balance of the influence of optical (Owsley, 2011) versus neural factors (Faubert, 2002) in the determination of functional performance level. Modifications occurring in the visual cortex, as well as more generally in the functional organisation of visual processing, could, therefore, be responsible for age related changes in visual function (Cabeza et al., 1997; Grady, 2008) not explained by psychophysical measures such as visual acuity and contrast sensitivity and could potentially be captured by self-reports. In fact, one of the most notable changes in cognitive ageing is the decline in visual processing speed that is also a marker of cognitive decline and brain dysfunction (Salthouse, 1996; Salthouse et al., 1996). Visual search is also one of the domains of visual processing that deteriorates with ageing in a manner that relates to visual attention decline (Madden, 2007). The natural decline in visual processing has a number of consequences on the ability to perform daily living tasks such as navigating the environment (including the possibility of suffering a fall) (Anstey et al., 2009), looking for a particular object or a person among others (Sekuler & Ball, 1986) and driving (Ball et al., 2006). These processes also mediate the successful training of visual capacities to improve daily living activities (Ball, Edwards & Ross, 2007; Edwards et al., 2013) and could, therefore, impact self perceived quality of vision. In the present study we assessed visual processing speed by the Choice Response Time Task, a specifically devised task based on the vast literature on processing speed tasks (see Salthouse, 1991), and visual search and attention as assessed by the Trail Making Test, a commonly used neurocognitive test (D’Elia et al., 1996). This test was selected on the basis that visual search, scanning and visual attention are engaged in this task (Sanchez-Cubillo et al., 2009). However it differs from typical visual search tasks in which a target present/target absent response is required and the features and number of distracters are manipulated (Treisman & Gelade, 1980; Wolfe, 1998). Working memory and planning are strongly involved in this task, as well as a motor component which is usually absent in visual search tasks (Sanchez-Cubillo et al., 2009).

A number of other factors need to be controlled for that may determine self reported vision: eye disease is common in older adults, and can result in substantial visual loss not captured by visual acuity tests (Charalampidou et al., 2011); the awareness of a visual problem could potentially be linked to the frequency of attendance for eye health examination, which should be accounted for. These vision-related factors were controlled for in the present study (see ‘Method’). In addition socio-demographic and general physical and mental health factors, as well as global cognitive status were accounted for as they could co-determine the self reports (e.g., Hidalgo et al., 2009).

## Materials & Methods

### Sample

Data from the first wave of the nationally representative Irish Longitudinal Study on Ageing (TILDA) were used for this study (*N* = 8,175 aged 50 years and older). The recruitment and sampling method has been described elsewhere (Kearney et al., 2011). Ethical approval was obtained from the Trinity College Dublin research ethics committee, and all participants provided written informed consent in accordance with the Declaration of Helsinki.

All participants completed a Computer Assisted Personal Interview (CAPI) in their own home, and each was then invited to a complete health assessment (Kearney et al., 2011). For the purpose of this study, only participants who attended the health assessment centre were included (*N* = 5,026; 2,304 male). The time delay between the CAPI interview and the health assessment was approximately three months. The question relating to self-reported visual acuity was asked in the CAPI, the cognitive tasks utilized in this study as well as the visual acuity and contrast sensitivity tests were administered in the health assessment. Those respondents who reported themselves as registered blind (legally defined as visual acuity less than LogMAR 1.0 (6/60) or visual field less than 20°) were also excluded from all the analyses (*N* = 5, 0.1% of the sample). The total sample size, therefore, included in the current study was *N* = 5,021 (2,299 male).

### Outcome variable

Self Reported Vision (SRV) was assessed during the CAPI with the following question: “Is your eyesight (using glasses or contact lenses if you need them) … excellent, very good, good, fair, poor, registered blind”. Those who reported themselves as ‘registered blind’ were not included in the study yielding a five point ordered scale for SRV.

### Predictor variables

Choice Reaction Time (CRT) test: the CRT is a computer based test, and was administered at the health assessment according to a Standard Operating Procedure. The task consisted of three phases: maintaining the starting button press; releasing the button at the appearance of the target stimulus (words “yes” or “no”) on the computer screen (cognitive speed), and finally, pressing the button corresponding to “yes” or “no” on the button-box (motor speed). Instructions emphasized accuracy as well as speed. Response times from target onset to the release of the starting button indicated the ‘cognitive’ (decision) time and the time taken to press the ‘yes’ or ‘no’ button was taken as ‘motor’ response time.

### Trail Making Tests

In TILDA, the Trail Making Test (TMT) utilized is the colour version, which is considered less biased by literacy and cultural differences (D’Elia et al., 1996). The task is administered in two versions, the first is simpler, requiring to draw a line between consecutive numbers (1–25) distributed on a sheet of paper (TMT1), and the second is more complex, requiring to draw a line alternating between numbers of two different colours (TMT2). This test was also administered during the Health Assessment following a Standard Operating Procedure. Before each of the TMTs, respondents were allowed a short practice run. The total time to perform the TMT (1 and 2) task, as recorded by the nurse with a stopwatch, was used for the current study. The nurses were trained to follow the Standard Operating Procedures and their performance was subjected to quality control according with the TILDA training protocols.

### Covariates

Visual acuity was measured monocularly during the health assessment using a LogMAR chart, and ETDRS letterset, at a test distance of four meters under photopic illumination conditions. Subjects were required, using their habitual refractive correction where applicable, to identify the letters presented on the chart in decreasing size (0.1 log unit decrease in size between lines). Visual acuity was recorded as the smallest line of letters accurately identified, accounting for any incorrectly identified letters on this line (each line consisted of five letters, each assigned a value of 0.02 log units, such that if a subject, for example, correctly identified four letters of the LogMAR 0.1 line, and incorrectly identified one letter, the final acuity was recorded as 0.12). For the present study visual acuity in the best performing eye was considered.

Contrast sensitivity (CS) was measured monocularly, with distance spectacles worn (where appropriate), using the Functional Vision Analyser™ (FVA; Stereo Optical Co., Inc—Chicago, USA). For the methodology see Loughman et al. (2012). Testing was performed under mesopic (3 cd/m2) background illumination conditions.

Covariates considered in this study were vision-related factors such as ocular pathology and ophthalmology/optometrist visits and other covariates related to demographic factors and physical and psychological health.

Eye disease was self reported during the CAPI interview. Respondents were asked to indicate the nature of any existing doctor-confirmed diagnosis of eye disease. Subjects were additionally asked to provide information in relation to their habitual use of spectacles or contact lenses, and visits to an optometrist.

Other covariates considered in this study were: age, sex, education, health (chronic conditions), wealth (based on house value), all of which were self-reported; hypertension (seating position systolic blood pressure > 140 ml mercury or diastolic > 90 ml mercury) and depression (Centre for Epidemiological Studies Depression Scale, CES-D), all of which were measured during the health centre visit. Global cognitive status was assessed by the Montreal Cognitive Assessment, (MoCA), a widely used neurocognitive assessment. For the purpose of the present work we excluded the visual items to avoid a bias against individuals with poor vision onto the overall cognitive assessment (see Cronin-Golomb et al., 2007). Table S2 summarises the variables utilized.

### Statistical analysis

Ordinal logistic regression was used to investigate the association between the variables of interest (CRT and TMT) and the outcome variable (self-report of vision on a five point Likert scale). Ordinal logistic regression yields odds ratios that can be interpreted in the same way as for binary logistic regression, but irrespective of the dichotomization of the outcome variable. The ‘proportional odds’ assumption is therefore key; this is the assumption that ORs generated by the ordinal regression are applicable across the whole of the response range of the outcome. Each of our variables of interest passed a ‘Brant’ test for proportional odds (Brant, 1990) (using the ‘brant’ command in Stata) and so this assumption was met. Univariate odds ratios were estimated for each predictor, as well as odds ratios adjusted for all covariates and other predictors.

Inverse probability weights were estimated and applied to all analyses to ensure that results based on the sample included in the current analysis were applicable to the community living middle aged and older population of Ireland. Statistical analyses were performed using STATA version 12 (StataCorp LP, College Station, TX).

## Results & Discussion

Using the legal (visual acuity based) definition of visual impairment (corrected visual acuity <20/40 or >0.3 LogMar) as a proxy indicator of good (equal or better than 0.3) versus poor (worse than 0.3) visual acuity, 89.8% of participants who reported good vision (excellent to good) had good visual acuity, while the remaining 10.2% demonstrated poor visual acuity. Among respondents rating their vision as poor, only 22.4% showed impaired visual acuity.

Considering the exclusion of participants presenting missing values on at least one of the variables of interest or covariates, the total sample entered into the final model was *N* = 4,178 (1,937 male), of which, 292 individuals (7%) reported fair or poor vision.

The descriptive statistics of the sample are presented in Table 1.

**Table 1:** Characteristics of the sample (*N* = 4,178 weighted to represent the population).

Variable	Excellent vision	Very good vision	Good vision	Fair vision	Poor vision	*P* value ^a^
Trail Making Test 1 (s)	2.81 (2.70–2.91)	3.01− (2.93–3.1)	3.15 (3.06–3.24)	3.45 (3.23–3.66)	4.18 (3.7–4.66)	<0.001
Trail Making Test 2 (s)	2.77 (2.66–2.87)	3.04 (2.95–3.13)	3.23 (3.14–3.33)	3.45 (3.25–3.66)	4.22 (3.76–4.69)	<0.001
CRT total time (ms)	787.18 (767.25–807.10)	823.97 (806.58–841.36)	836.29 (817.4–855.18)	904.14 (829.28–979)	1016.75 (827.64–1205.86)	<0.001
CRT cog time (ms)	522.77 (509.72–535.81)	538.85 (528.74–548.95)	543.72 (533.81–553.63)	582.69 (546.49–618.89)	648.5 (545.80–751.19)	<0.001
CRT mot time (ms)	265.95 (256.47–275.44)	287.61 (278.62–296.61)	294.33 (283.41–305.24)	321.88 (281.16–362.61)	371.46 (281.06–461.85)	<0.001
MoCA score (/30)	25.48 (25.25–25.71)	24.96 (24.76–25.16)	24.54 (24.29–24.8)	23.8 (23.27–24.32)	21.80 (20.24–23.36)	<0.001
Visual reasoning (/5)	3.18 (3.09–3.28)	3 (2.92–3.07)	2.86 (2.77–2.96)	2.90 (2.71–3.09)	2.69 (1.74–3.64)	<0.001
Visual acuity (LogMar)	.025 (.01–.04)	.061 (.05–.07)	.09 (.07–.1)	.12 (.1–.15)	.26 (.14–.37)	<0.001
Contrast sensitivity low fq	38.19 (36.59–39.8)	36.6 (35.5–37.7)	36.45 (35.17–37.73)	33.84 (30.96–36.71)	22.23 (14.43–30.03)	<0.001
Contrast sensitivity middle fq	52.72 (50.92–54.52)	49.56 (48.03–51.09)	48.12 (46.51–49.74)	43.19 (39.55–46.83)	27.98 (19.39–36.58)	<0.001
Contrast sensitivity high fq	4.29 (3.87–4.71)	3.54 (3.22–3.86)	3.23 (2.92–3.54)	3.07 (1.56–4.59)	1.13 (−.06–2.32)	<0.001
Ocular pathology (any)	8.4 (6.2–11.3)	13.2 (11.1–15.5)	18.3 (1.6–21.3)	32.41 (25.72–4)	67.4 (46.5–83.2)	<0.001
Cataract	5.9 (4.1–8.6)	9 (7.2–11.2)	11.7 (9.4–14.5)	21.02 (14.9–28.9)	41.5 (20.7–65.9)	<0.001
Glaucoma	.6 (.3–01)	1.7 (1.13–2.6)	2.4 (1.7–03.6)	5.5 (2.8–10.5)	13.3 (3.2–41.8)	<0.001
AMD	.7 (.3–1.6)	.92 (.5–1.7)	1.9 (1.1–3.2)	3.7 (1.4–9.1)	17.7 (4.8–48)	<0.001
Other	1.24 (.7–2.2)	1.96 (1.28–3.02)	3.66 (2.8–4.8)	6.1 (3.79–9.8)	8.8 (2.78–24.7)	<0.001
Wear glasses	68.8 (64.6–72.6)	63.5 (60.1–66.7)	61.5 (57.6–65.3)	62.2 (55.2–68.7)	59.1 (35.3–79.3)	0.177
Visited optometrist	8.8 (6.6–11.6)	13.9 (11.7–16.4)	13 (10.8–15.5)	19.05 (13.21–26.7)	36.2 (17–61.2)	<0.001
Wealth (rent or house value <400.000 euro)	76.2 (72.1–79.9)	83.8 (81.2–86.1)	87.3 (84.8–89.4)	90.6 (86.25–93.7)	90.2 (76. 1–96.4)	<0.001
Age	61.45 (60.70–62.19)	62.81 (62.14–63.48)	63.13 (62.38–63.88)	63.35 (61.61–65.09)	69.39 (64.92–73.87)	<0.001
Sex (% male)	51.7 (48.2–55.3)	48.4 (45.8–51.1)	48.2 (45.2–51.2)	48.1 (40.9–55.4)	39.4 (21.3–60.9)	0.5673
Education primary or none	22.5 (19–26.4)	32.8 (29.8–36)	38.4 (34.9–42.1)	49.23 (42.1–56.4)	70.8 (52.3–84.2)	<0.001
Hypertension^b^	38.4 (34.7–42.2)	42.7 (39.8–45.8)	40.15 (37–43.4)	43.6 (36.3–51.1)	65.1 (43–82.2)	<0.05
Stroke or TIA	1.4 (.8–2.4)	2.2 (1.54–3.3)	4.9 (3.53–6.7)	6.1 (2.92–12.3)	4.8 (1.1–19.3)	<0.01
Diabetes	5.5 (3.9–7.8)	6.5 (5.1–8.1)	7.7 (6.1–9.7)	6.1 (3.5–10.2)	9.5 (3.2–25)	0.53
Chronic conditions	47.4 (43.6–51.1)	51.5 (48.6–54.4)	56.3 (53.1–59.4)	71.9 (65.4–77.6)	76.3 (58.3–88.1)	<0.001
Hearing (poor)	1.23 (.63–2.4)	1.1 (.66–1.8)	2.58 (1.8–3.7)	4.1 (2–8.2)	13.6 (3.6–40.2)	<0.001
Depression (severe)	6.1 (4.4–8.3)	7.9 (6.45–9.7)	9.5 (7.7–11.6)	17.3 (12.4–23.5)	27.7 (12.3–51.1)	<0.001

**Notes.**

a The *P* values refer to one-way ANOVA tests for means comparison or Chi-square tests for frequencies comparisons. Confidence intervals in parentheses.

b All cardiovascular health measures are self-reported (CAPI interview) apart from Hypertension that is measured in the heath assessment.

The odd ratios for the association between each predictor variable and poor SRV are shown in Table 2.

**Table 2:** Ordinal odds ratios representing the effect of potential contributing factors to self- reported vision (excellent, very good, good, fair, poor). Participants suffering from Dementia, Parkinson’s Disease and serious memory impairment (all by self-report) were excluded from the analysis because they present cognitive impairment or neurologic illness that could influence the relationship between cognition and self-reported vision. Analyses are adjusted for clustered sample design and sampling weights.

	Univariate effect		Multivariate effect	
	OR	95% CI	OR	95% CI
Trail making task time (best quintile)	1	[1.00, 1.00]	1	[1.00, 1.00]
2nd	0.96	[0.61, 1.52]	0.96	[0.79, 1.16]
3rd	1.19	[0.77, 1.83]	1.06	[0.86, 1.30]
4th	1.55^*^	[1.02, 2.34]	1.05	[0.83, 1.33]
5th quintile	3.16^***^	[2.11, 4.71]	1.36^*^	[1.05, 1.76]
Choice reaction task time (fastest)	1	[1.00, 1.00]	1	[1.00, 1.00]
2nd	1.43	[0.92, 2.22]	1.09	[0.90, 1.31]
3rd	1.2	[0.76, 1.90]	0.97	[0.80, 1.18]
4th	1.56	[0.99, 2.44]	1.1	[0.89, 1.38]
Slowest quintile	3.00^***^	[1.91, 4.71]	1.21	[0.95, 1.54]
Visual acuity (best quintile)	1	[1.00, 1.00]	1	[1.00, 1.00]
2nd	1.60^*^	[1.01, 2.56]	1.2	[0.99, 1.46]
3rd	1.89^**^	[1.28, 2.81]	1.30^*^	[1.06, 1.61]
4th	3.13^***^	[2.10, 4.66]	1.52^***^	[1.22, 1.89]
Lowest quintile	4.02^***^	[2.70, 5.99]	1.72^***^	[1.35, 2.18]
High frequency CS (lowest quintile)	1	[1.00, 1.00]	1	[1.00, 1.00]
2nd	1.37	[0.93, 2.02]	0.86	[0.69, 1.07]
3rd	1.79^**^	[1.19, 2.69]	1.08	[0.86, 1.36]
4th	0.8	[0.52, 1.22]	1.03	[0.83, 1.27]
Highest quintile	0.83	[0.54, 1.27]	1.03	[0.83, 1.28]
Low frequency CS (lowest quintile)	3.09^***^	[2.03, 4.71]	1.22	[0.97, 1.54]
2nd	1.21	[0.76, 1.95]	1.02	[0.84, 1.25]
3rd	1.4	[0.92, 2.12]	1	[0.81, 1.23]
4th	1.13	[0.70, 1.82]	0.99	[0.81, 1.20]
Highest quintile	1	[1.00, 1.00]	1	[1.00, 1.00]
Any diagnosed eye pathology	4.04^***^	[3.02, 5.41]	2.25^***^	[1.77, 2.87]
Doesn’t wear glasses	1.12	[0.86, 1.47]	1.32^***^	[1.12, 1.55]
Optician visit	1.73^**^	[1.20, 2.49]	1.01	[0.80, 1.27]
MoCA score without visual elements (per point)	0.89^***^	[0.86, 0.92]	0.98^*^	[0.95, 1.00]
Female vs male	1.18	[0.91, 1.54]	0.99	[0.86, 1.14]
Primary education (ref)	1	[1.00, 1.00]	1	[1.00, 1.00]
Secondary education	0.42^***^	[0.32, 0.55]	0.70^***^	[0.59, 0.83]
Tertiary or higher education	0.33^***^	[0.24, 0.44]	0.64^***^	[0.53, 0.77]
Total Wealth (>€ 400,000)	0.44^***^	[0.31, 0.61]	0.71^***^	[0.59, 0.85]
Age 50–54	1	[1.00, 1.00]	1	[1.00, 1.00]
55–59	0.88	[0.62, 1.24]	0.9	[0.74, 1.09]
60–64	0.94	[0.64, 1.38]	0.78^*^	[0.63, 0.95]
65–69	1.07	[0.75, 1.54]	0.67^***^	[0.52, 0.85]
70–74	1.08	[0.71, 1.66]	0.64^**^	[0.48, 0.84]
75–79	1.59	[0.94, 2.70]	0.8	[0.56, 1.14]
80+	3.27^***^	[1.84, 5.79]	0.68	[0.37, 1.28]
Hypertension	0.9	[0.65, 1.23]	0.99	[0.84, 1.16]
Diabetes	1.21	[0.75, 1.96]	0.95	[0.70, 1.28]
Stroke	2.07^*^	[1.07, 3.99]	1.83^**^	[1.22, 2.75]
Not depressed (CES-D <8)	1	[1.00, 1.00]	1	[1.00, 1.00]
Sub-threshold depressive symptoms (CES-D 8-15)	2.14^***^	[1.57, 2.92]	1.70^***^	[1.41, 2.04]
Significant depression (CES-D ≥16)	3.89^***^	[2.71, 5.56]	1.96^***^	[1.42, 2.70]

**Notes.**

* *p* < 0.05.

** *p* < 0.01.

*** *p* < 0.001.

There was initial evidence that poor processing speed as measured by CRT independently influenced SRV, but this was not statistically significant (O.R. = 1.21; *p* > 0.05 for poorest compared to best quintile). There was a dose–response relationship between slow TMT and SRV in univariate analysis. After adjusting for all other factors, those participants with visual search times in the slowest quintile had poorer SRV (OR = 1.36; *p* < 0.05). It is worth noting that the question on SRV was answered several weeks before the health assessment, therefore, carry-on effects from the cognitive tasks to the self-reports can be likely excluded.

As expected there was a gradient in SRV with worsening visual acuity, with odds ratio of 1.72 for lowest quintile (corresponding to LogMar mean = .345, standard deviation = .153) compared to the highest quintile (LogMar mean −.131, standard deviation = .054). The univariate effect of low-frequency CS (OR = 3.1; *p* < 0.01 for poorest compared to highest quintile) was almost completely explained by confounding factors in the fully adjusted model. High frequency CS was not related to SRV independently of visual function.

As to other vision-related covariates, eye disease was associated with self reporting poor vision (OR = 2.25, *p* < 0.001) not wearing glasses had a small but significant impact in increasing the odds of poor SRV (OR = 1.32; *p* < 0.001).

As to the other covariates, age, education and wealth were significant determinants of the self reports. Global cognitive function measured by MoCA (with visual elements removed) retained a small statistically significant effect on SRV indicating a contribution of general cognitive function even after adjusting for all other covariates (OR = 0.98 per point; *p* < 0.05).

Mental health had a highly significant impact on the ratings, with a dose response pattern indicating that more depressed participants were more likely to rate their vision as poorer. In addition depression may have affected SVR due to a general decrease in the ability to carry out daily functioning, in line with the finding that self-reports are modulated by health status (e.g., Ponds, Van Boxtel & Jolles, 2000).

To clarify whether the relationship of TMT with SRV performance could be attributed to visual processing skills in this context, more so than to global cognitive function, additional analyses were performed. Adding to the model individual MoCA subscores related to working memory (serial subtraction) and executive function (verbal fluency), which have been associated with TMT performance (Crowe, 1998) or visual reasoning (indicative of fluid intelligence) (Salthouse, 2011) did not attenuate the effect of TMT on SRV (data not shown).

It is worth noting that the prevalence of sub-optimal self reported visual function (identifiable in ‘fair’ and ‘poor’ vision) was relatively low (7%). However this is in line with existing literature, for example a prevalence of self reported fair and poor vision of 10% in people aged between 40 and 69 was reported (Klein et al., 1999), while the prevalence increased over 70 years of age (Hidalgo et al., 2009) reported a prevalence of 6% (bad or very bad vision by self report) in participants age 65 and older. Although the current study confirms a relationship between visual acuity and SRV, additional factors including the presence of ocular pathology and reduced visual processing capacity are identified as important determinants of SRV.

Visual processing, as assessed TMT was associated with SRV, independent of global cognition. The lack of significance of the CRT in the fully adjusted model was to be expected, in consideration of the fact that processing speed for visual targets was also captured by the TMT. The association between slow TMT and poor SRV may indicate that the TMT captures the contribution of visual search and visual attention (Crowe, 1998; Salthouse, 2011; Sanchez-Cubillo et al., 2009) to SRV. Indeed TMT has been used previously as a measure of visual scanning to predict driving restriction or cessation in line with other visual attention tasks (Keay et al., 2009). However, TMT also captures visuo-motor skills also declining with age, therefore it is not possible to exclude that decline in the ability to trace the trail may have influenced the results. Nonetheless, the lack of significant association between the CRT (cognitive and motor components) to SRV supports the idea that the visual processing component of TMT is more salient than the motor component in determining the association between TMT and SRV.

As expected, visual acuity and (self reported) ocular pathologies, independent of VA and CS, were associated with a substantial increase in the likelihood of reporting poor vision. The increased level of association suggests that self-reports capture day to day visual deficits that are not fully captured by acuity measures (Charalampidou et al., 2011; Tejeria et al., 2002).

The contribution of wearing glasses to self-reports could be due to a need for refractive correction, whereby respondents not wearing glasses may need to correct a deficit that is not entirely captured by the distance visual acuity test, for example, for near vision.

Cognitive status, assessed by the MoCA indicated that higher cognitive scores were associated with decreased likelihood of self reporting poor vision. This result could be interpreted in terms of coping strategies. It is plausible to assume that memory and attention could contribute in maintaining functional efficiency in spite of a visual acuity deficit. It has been indicated previously, that the Short Portable Mental Status Questionnaire score (dichotomized according impairment and no impairment), which assesses global cognition, was associated with the score obtained in the Visual Function Index questionnaire (VF-14) (Hidalgo et al., 2009).

Among the other covariates, the protective effect of age was notable, with older olds (65+) reporting relatively better vision than younger olds (50–54) when acuity was controlled for. This may suggest a positive bias in self reports with ageing. Alternatively it may indicate that older people have developed coping strategies that alleviate the impact of their visual deficits on their daily life.

Higher educated individuals and wealthier individuals were also more likely to report good vision. This may be due to a positive attitude in self reports. However at present it is not possible to exclude that both self reported health and quality of life may be related to better coping strategies developed by more educated and wealthier individuals.

The effect of depression can plausibly be attributed to a general tendency of depressed individuals to a pessimistic view on their health. Alternatively, or complementarily, it is possible that poorer vision is associated with depression due to the withdrawal typically experienced by depressed individuals, which, in turn, is associated with cognitive and, possibly, perceptual decline (Austin, Mitchell & Goodwin, 2001).

## Conclusions

Our results confirm the existing evidence that visual acuity is a clear contributor to SRV as well as ocular pathology. Other factors such as depression are significant determinants of SRV. More importantly in relation to our hypotheses, higher level visual processing, in terms of response time to visual stimuli (CRT/TMT) and time to perform a task requiring visual search (TMT) play a part in SRV. This may be due to visual deficits linked to higher level visual processing that are not fully captured by psychophysical tests. A limitation of this study is constituted by the time delay between the CAPI and the health assessment, whereby the SRV question was always asked before (approximately three months) the vision and cognitive tests were administered. Therefore we cannot completely exclude biases due to the order of testing. However we feel that considering the vast amount of questions asked in the CAPI and the intensive assessment occurring in the health assessment it is unlikely that expectations on specific tests and their associations could be formed and significantly influence performance. A limitation of this study is also that TMT is not the ‘purest’ instrument to test the contribution of higher level visual processing to SRV because of the nature of the visual search required by the task and the variety of cognitive processes involved in performing it, especially working memory and attention (Crowe, 1998; Salthouse, 2011; Sanchez-Cubillo et al., 2009). Experimental tasks testing correlations between self reported vision and a wider range of visual processing skills going from psychophysical measures, to visual discrimination, to visual search, to more cognitively loaded vision based tasks such as TMT would allow determining which aspects of visual cognition underlie the apparently simple fact that one individual judges her/his vision as excellent and another poor. Such an experimental approach would complement the epidemiological approach proposed here, where only a limited amount of assessments could be administered. CRT and TMT represent viable instruments in the context of this epidemiological study aimed at assessing a broad spectrum of health related variables. Indeed epidemiological studies have shown that SRV is associated with physical and cognitive limitations and decline (e.g., Rogers & Langa, 2010), the present study adds to this literature by showing that poorer SRV can be associated not only with poor visual acuity but also with poorer cognitive abilities related to visual processing, which are in turn likely to influence the capability to cope with visual acuity deficits themselves.

## Supplemental Information

10.7717/peerj.1407/supp-1 Table S1 Overview of literature Overview of studies comparing self-reports of vision and objective measures, in particular those taking into account cognitive and socio-demographic variables.

10.7717/peerj.1407/supp-2 Table S2 Measures utilised in the study Variables from the TILDA study utilised in the present study.

## Additional Information and Declarations

### Data Availability

The following information was supplied regarding data availability: Irish Social Science Data Archive: http://www.ucd.ie/issda/data/tilda/.
