# Supplementary material for: Trail Making Test performance contributes to subjective judgment of visual efficiency in older adults
Source: PeerJ. 2015 Dec 7;3:e1407. doi: 10.7717/peerj.1407 (PMC4675109; doi:10.7717/peerj.1407)
Supplement: Table S1 — Overview of studies comparing self-reports of vision and objective measures, in particular those taking into account cognitive and socio-demographic variables. [file peerj-03-1407-s001.docx]

Table S1. Overview of studies comparing self-reports of vision and objective measures, in particular those taking into account cognitive and socio-demographic variables.

| **Study and Population** | **Psychophysical Vision Measures** | **Self-reports of Vision** | **Cognitive, Socio-demographic, Health variables** | **Relationship between variables** |
| --- | --- | --- | --- | --- |
| (Klein, 1999)  Beaver Dam Eye Study (age 40+ ) | Distance (current and best corrected) and near visual acuity;  Contrast sensitivity;  Visual sensitivity (visual field) | General (excellent, very good, good, fair, poor)  Specific domains: reading small and regular print; reading road signs; recognising people/objects across the street; night driving [all referred to corrected vision] | Socio-demographic: Age  Sex | Correlation between psychophysical distance vision and general self report no report of sex or age difference in strength of correlation |
| (Eekhof, 2000)  GP practices (age 75+) | Distance (corrected) and near visual acuity | Difficulty recognising a face; difficulty reading letters in newspaper | None | 79% and 69% concordance respectively between objective and subjective measures |
| (Rubin, 2001) SEE Project (age 65-84) | Distance visual acuity; contrast sensitivity; glare; stereoacuity; visual field | Activity of Daily Vision Scale (also general question) | Socio-demographic: age, race, sex, education,  Cognition: MMSE,  Health: comorbidities, depression | Acuity and contrast were associated with highest odds of reporting poor visual function after controlling for all other variables. |
| (Kempen, 1996)  sub-sample of the Groningen Longitudinal Ageing study (age 57+) | Distance visual acuity, edge contrast sensitivity, disability glare, text reading and foveal light sensitivity | Use of vision (e.g. can you see approaching cars and bicycles in the street? Can you read numbers in a phonebook?) with multiple choice answers (‘(almost)always; often; sometimes; (almost)never’) | Socio-demographic: age, sex, education  Cognition: short term memory test (visual presentation; immediate recall delayed recall and recognition) and the Adults Reading Test (NART)  Health: anxiety, depression, personality, mastery/control | Independent predictors after controlling for vision measures: age, education,  NART, anxiety |
| (Hidalgo, 2009 )  1387 residents of the city of Albacete (Spain) (age 65+) | Visual acuity | Visual Function Index questionnaire and general self report (from very good to very poor) | Socio-demographic: age, sex, education, marital status, social class  Cognition: Short Portable Mental Status Questionnaire  Health: chronic diseases; activities of daily living dependence; medicine consumption; depression | Independent predictors of the Visual Function index:  Visual acuity, self-reported vision (general question), age, being female, dependence in activities of daily living, cognition, depression |
