# Supplementary material for: Trail Making Test performance contributes to subjective judgment of visual efficiency in older adults
Source: PeerJ. 2015 Dec 7;3:e1407. doi: 10.7717/peerj.1407 (PMC4675109; doi:10.7717/peerj.1407)
Supplement: Table S2 — Variables from the TILDA study utilised in the present study. [file peerj-03-1407-s002.docx]

Table S2. Variables from the TILDA study utilised in the present study.

| **Task (when assessed)** | **Instructions** | **Ability assessed** | **Scoring (used in the present work)** |
| --- | --- | --- | --- |
| **Outcome variable:** | | | |
| Self reported vision (CAPI) | ‘Is your eyesight (using glasses or contact lenses if you use them)...Excellent, Very Good, Good, Fair, Poor’ | Vision (self reported) | 1 to 6 (Excellent to registered blind), only 5 point scale used here (registered blind participants excluded) |
| **Predictors:** | | | |
| Choice Response Time test, CRT (HA) | Computer based task requiring speeded response to a stimulus appearing on screen (yes/no). | Processing speed | Response time (RT, ms) |
| Trail Making Test, TMT (1) and (2) | (1) to draw a line between a sequence of circles numbered 1 to 25;  (2) same as 1 but alternating between numbers of 2 different colours | Visual scanning, visual attention, executive function | Time taken to perform the task (sec) |
| **Covariates:** | | | |
| Visual acuity (LogMar) (HA) | Monocular test.  LogMar chart positioned on a wall mounted cabinet (4mt distance from the respondent). Read each letter on the chart starting from the top left. Guess when uncertain about the letter | Visual acuity | LogMar score according to SOP (Respondents presenting particular difficulty in the test were allowed to decrease the distance from the chart to 1mt, in this case their score was increased by 0.6 LogMar) |
| Contrast sensitivity (HA) | Measured with the Functional Visual Analyzer™ by a technique called functional acuity contrast test (F.A.C.T.®) | Contrast sensitivity | A score is assigned for the maximum level of contrast at which the respondent was able to identify the orientation of the gratings for each spatial frequency |
| Eye disease (CAPI) | ‘Has your doctor ever told you that you have any of the following eye diseases..? Cataract, glaucoma, AMD, other.’ | Eye disease (self report) | At least one vs. none |
| Wearing glasses (CAPI) | ‘Do you usually wear glasses or contact lenses? Yes, No. [usually it was intended most of the time]’ | Wearing glasses (self report) | Yes vs. no |
| Health care utilization, optician | ‘In the last 12 month did you receive any of the following state services?...optician’, [only free state provided services] | Visited an optician | Yes vs. no |
|  |  |  |  |
| Age | Age (assuming that day of birth is first day of the month) | Age | Categorized in 5 years brackets from to 80 and 80+ |
| Wealth (CAPI) | House value (multiple choice) (due to high number of missing cases in the income variable N=4381 vs. N=500 for house value) | Economic status | Dichotomised in  1) <=400.000  2)>400.000 |
| Education (CAPI) | Question on highest education achieved | Education | Primary or none  Secondary  Tertiary |
| Blood pressure (hypertension) (HA) | Hypertension: seating position systolic blood pressure > 140 ml mercury or a seated position diastolic blood pressure > 90 ml mercury | Hypertension | Yes/no |
| Stroke, transient ischemic attack (TIA) and diabetes non heart related chronic conditions (lung disease, asthma, arthritis, osteoporosis, cancer, stomach ulcers, varicose ulcers, cirrhosis) (CAPI) | ‘Has a doctor ever told you that you have any of the conditions on this card [name conditions]...?’ | Heart and not heart related chronic conditions | At least one vs. none |
| Centre for Epidemiological Studies Depression Scale (CES-D) (CAPI) | Questionnaire | Depression | 0-7 no depression or mild; 8 to 15 moderate; 16 to 60 severe depressive symptoms |
| MoCA (HA)^ | Questionnaire on different aspects of cognition | Visuo-spatial/executive, language; memory, attention, reasoning, orientation | Score on 30 points |

^ vision-based items of the Montreal Cognitive Assessment were excluded from the score because individuals with poorer eyesight may have a disadvantage in those items that is not due to their cognitive fitness. Trail Making Test was excluded because it was introduced as specific assessment.
